# Cancer risk by the subtype of alopecia

**DOI:** 10.1038/s41598-018-28142-1

**Published:** 2018-06-27

**Authors:** Ji Hyun Lee, Yumee Song, Kyung Do Han, Young Min Park, Jun Young Lee, Yong-Gyu Park, Young Bok Lee

**Affiliations:** 1 Department of Dermatology, Seoul St. Mary’s Hospital, College of Medicine, The Catholic University of Korea, Seoul, Republic of Korea; 2 Department of Biostatistics, College of Medicine, The Catholic University of Korea, Seoul, Republic of Korea; 3 Department of Dermatology, Uijeongbu St. Mary’s Hospital, College of Medicine, The Catholic University of Korea, Seoul, Republic of Korea

## Abstract

The cancer risk in patients with alopecia areata (AA) or alopecia totalis (AT)/alopecia universalis (AU) remains unknown. In this study, national statistical data were used to study the association between these forms of alopecia and the risk of cancer. We enrolled 668,604 patients who were treated for alopecia from 2007 to 2014, and age- and sex-matched control subjects. AA and AT/AU patients had slightly higher overall cancer risks (hazard ratio (HR), 1.043; 95% confidence interval (CI), 1.022–1.065 and HR, 1.07; 95% CI, 1.013–1.129, respectively) than controls, after adjusting for confounding factors. The risks of oral cavity, esophagus, liver, biliary tract, pancreas, larynx, lung, kidney, breast, cervix, ovary, uterus, testis, nerve, and skin cancers; and lymphoma, multiple myeloma, and leukemia, were not increased in alopecia patients. In AA or AT/AU patients, the only increased risk was that of thyroid cancer. In AA patients alone, the risks of bladder and prostate cancers were increased. Thus, the cancer risks varied by the alopecia subtype. Careful monitoring is needed to explore if the actual risks of thyroid, bladder, and prostate cancers are increased in alopecia patients.

## Introduction

Alopecia areata (AA) is an inflammatory autoimmune disorder featuring temporary, non-scarring, patchy hair loss associated with genetic and environmental factors and T-cell-mediated immunological reactions. Alopecia totalis (AT) refers to total scalp hair loss, and alopecia universalis (AU) to hair loss over the entire body. AT and AU are poor prognostic forms of alopecia. Typical AA generally has a lifetime prevalence of about 2%^1^.

AA is associated with comorbidities such as psychiatric illnesses and autoimmune disorders. Associations between AA and autoimmune diseases including vitiligo, thyroid disease, irritable bowel syndrome, psoriasis, systemic lupus erythematosus, rheumatoid arthritis, and diabetes mellitus have been suggested. AA may be accompanied by depression and anxiety^2^. According to the recent World Health Organization Global Burden of Disease Study, the estimated disability-adjusted life years (DALY) lost by AA patients were higher than those lost by psoriasis patients^3^, particularly by patients with severe AA. The burden of AA is increasing, and some comorbidities have been reported; however, there have been few studies on cancer risks. Sun *et al*. reported that female patients with alopecia were at an increased risk of thyroid cancer^4^. However, this study was a small study, and the cancer risk remains unclear.

To date, national databases have not been used to explore possible associations between alopecia (total or by subtype) and cancers. In the present study, we explored the associations between various AA subtypes and cancer using a National Health Insurance System (NHIS) database.

## Results

### Baseline characteristics of the study population

**Figure 1:**
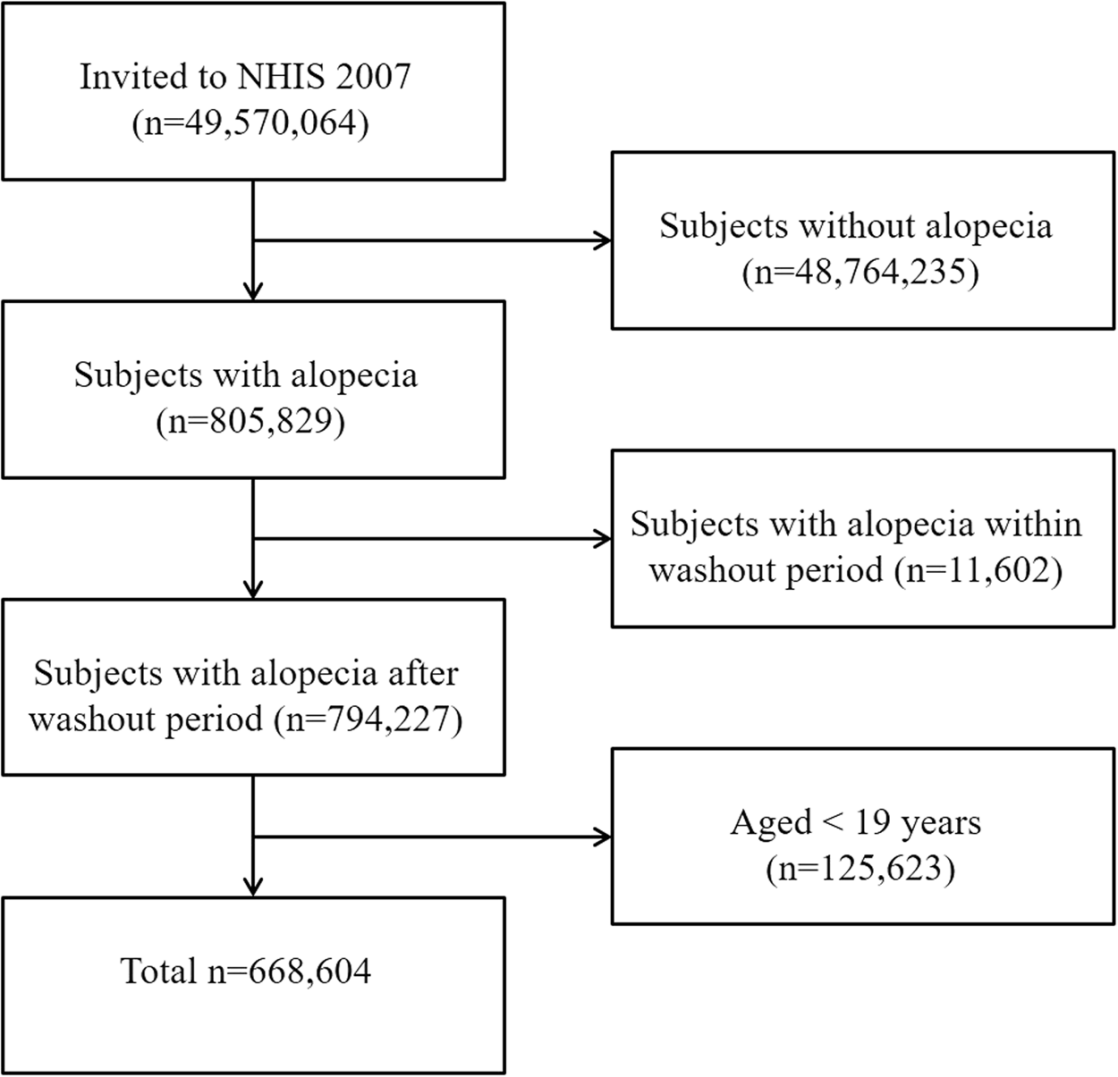
Flow diagram of AA patient selection.

In total, 668,604 patients were diagnosed with alopecia from 2007 to 2014. We included 608,190 patients with AA and 60,414 with AT/AU in the present study; follow-up ceased on December 31, 2015 (Fig. 1). The mean follow-up time was 4.92 ± 2.32 years. The demographic and clinical characteristics of the study population are shown in Table 1. The mean ages at diagnosis of AA and AT/AU were 40.16 ± 12.74 and 41.29 ± 13.86 years, respectively. The proportions of males with AA and AT/AU were 50.78% and 52.46%, respectively.

**Table 1:** Characteristics of the study population (controls and patients with alopecia).

	Alopecia	
	No	Yes
	(n = 3,343,020)	(n = 668,604)
Gender (male)	1,702,530 (50.93)	340,506 (50.93)
Age (years)	40.3 ± 12.8	40.3 ± 12.8
No. aged ≥ 65 years	154,640 (4.63)	30,928 (4.63)
Income level (lowest quartile)	772,480 (23.11)	147,947 (22.13)
Place of residence (urban)	1,570,224 (47.07)	320,668 (48.07)
Diabetes mellitus	122,315 (3.66)	24,115 (3.61)
Hypertension	318,889 (9.54)	66,187 (9.9)
Dyslipidemia	198,210 (5.93)	47,081 (7.04)

Values are expressed as n (%) or means ± SDs.

### Cancer risks based on the AA subtype

**Figure 2:**
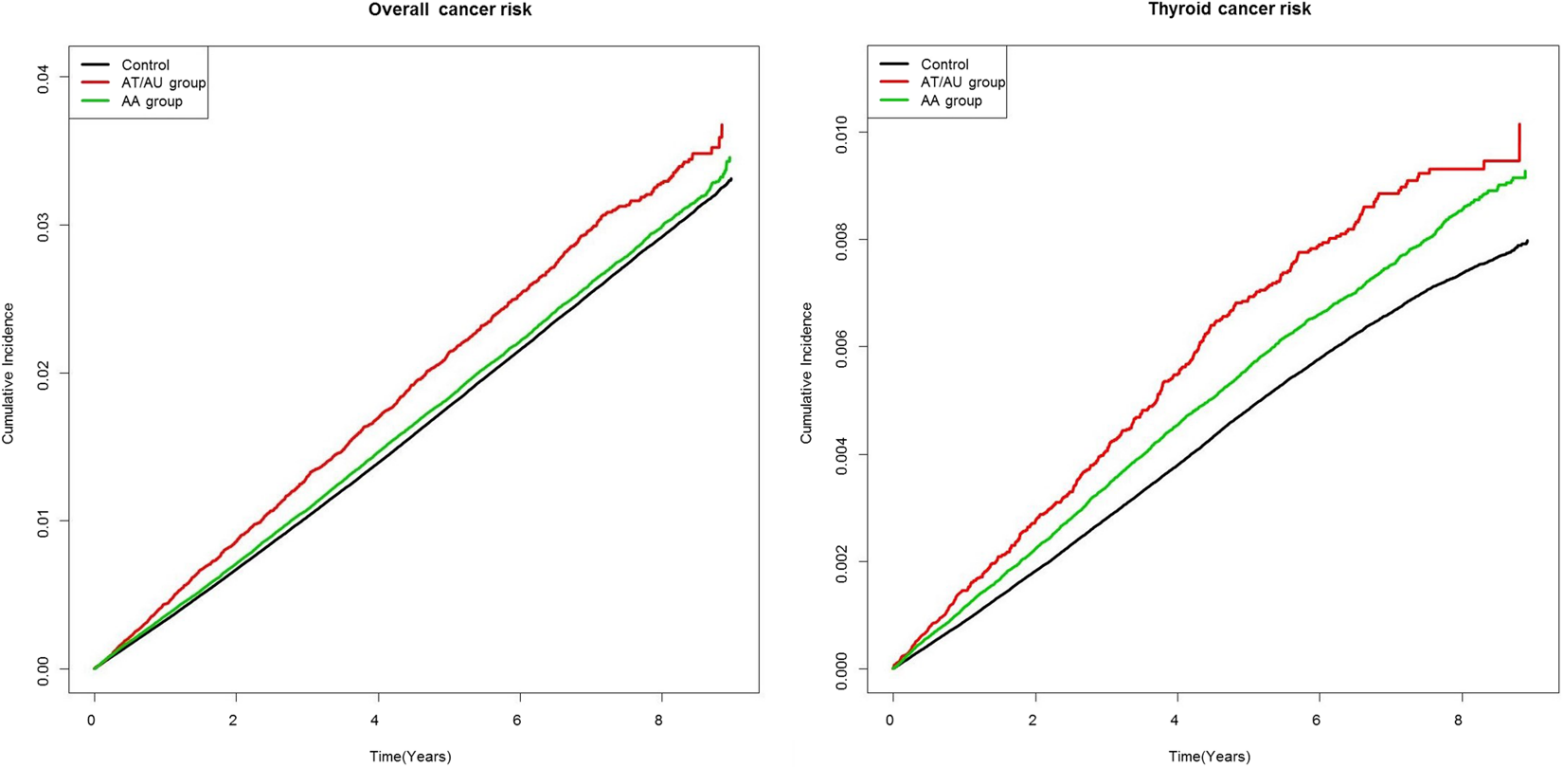
Cumulative incidences of overall and thyroid cancer in patients with different subtypes of alopecia (Kaplan-Meier modeling). (**a**) overall cancer, and (**b**) thyroid cancer. Control: Control subjects AA group: Patients with alopecia areata except alopecia totalis/alopecia universalis. AT/AU group: Patients with alopecia totalis/alopecia universalis.

Table 2 and Fig. 2 show the results obtained when the AA and AT/AU groups were analyzed by reference to ICD-10 codes. After adjusting for all confounding factors including age, gender, diabetes mellitus, hypertension, dyslipidemia, and income level, the overall cancer risk was higher in both the AA group (hazard ratio (HR), 1.043; 95% confidence interval (CI), 1.022–1.065) and the AT/AU group (HR, 1.070; 95% CI, 1.013–1.129). Notably, the risk of thyroid cancer was higher in the AT/AU group (HR, 1.332; 95% CI, 1.207–1.470) than the AA group (HR, 1.165; 95% CI, 1.122–1.210) or the control group. The risk of prostate cancer was significantly increased in the AA group (HR, 1.259; 95% CI, 1.139–1.391) compared with the AT/AU group (HR, 1.243; 95% CI, 0.963–1.604). In addition, the risk of bladder cancer was significantly increased in the AA group (HR, 1.216; 95% CI, 1.059–1.397) compared with the AT/AU group (HR, 1.024; 95% CI, 0.694–1.512). The risks for cancer of the oral cavity, esophagus, liver, biliary tract, pancreas, larynx, lung, kidney, breast, cervix, ovary, uterus, testis, nerves, and skin; and those for lymphoma, multiple myeloma, and leukemia, were not elevated in alopecia patients. Thus, neither AA nor AT/AU was associated with increased risks of cancers other than thyroid, prostate, and bladder cancers.

**Table 2:** Incidences of neoplasms in the control, AA, and AT/AU groups.

Group	Event	Crude IR	Multivariate Cox’s HR^a^
**Cancer**			
Control	59,339	3.611	1
AA group^b^	11,061	3.72798	1.043 (1.022,1.065)
AT/AU group^c^	1,349	4.25107	1.07 (1.013,1.129)
**Oral cavity**			
Control	912	0.054924	1
AA group	156	0.05201	0.953 (0.804,1.13)
AT/AU group	17	0.052875	0.873 (0.54,1.411)
**Esophagus**			
Control	440	0.026496	1
AA group	69	0.023002	0.882 (0.684,1.137)
AT/AU group	5	0.015549	0.505 (0.209,1.219)
**Stomach**			
Control	7,878	0.47504	1
AA group	1,317	0.43958	0.939 (0.886,0.996)
AT/AU group	156	0.48584	0.907 (0.774,1.063)
**Colorectal**			
Control	9,986	0.60229	1
AA group	1,653	0.55185	0.929 (0.882,0.979)
AT/AU group	217	0.67619	0.979 (0.856,1.12)
**Liver**			
Control	4,485	0.27026	1
AA group	764	0.25484	0.967 (0.896,1.044)
AT/AU group	74	0.23027	0.761 (0.605,0.958)
**Biliary tract**			
Control	1,094	0.065886	1
AA group	189	0.063012	0.974 (0.834,1.136)
AT/AU group	32	0.099541	1.205 (0.848,1.714)
**Pancreas**			
Control	2,815	0.16957	1
AA group	504	0.16807	1.007 (0.916,1.107)
AT/AU group	60	0.18668	0.936 (0.725,1.209)
**Larynx**			
Control	275	0.01656	1
AA group	46	0.015335	0.942 (0.689,1.287)
AT/AU group	2	0.00622	0.33 (0.082,1.326)
**Lung**			
Control	4,122	0.24837	1
AA group	753	0.25117	1.028 (0.951,1.111)
AT/AU group	95	0.29565	1 (0.816,1.226)
**Kidney**			
Control	1,346	0.08107	1
AA group	232	0.07735	0.949 (0.826,1.091)
AT/AU group	34	0.10576	1.164 (0.828,1.636)
**Thyroid**			
Control	15,554	0.93933	
AA group	3,276	1.09588	1.165 (1.122,1.21)
AT/AU group	406	1.26818	1.332 (1.207,1.47)
**Breast**			
Control	8,124	0.99083	1
AA group	1,430	0.96254	0.98 (0.926,1.036)
AT/AU group	184	1.19358	1.122 (0.969,1.298)
**Cervix**			
Control	1,910	0.23247	1
AA group	332	0.22301	0.97 (0.863,1.09)
AT/AU group	40	0.25872	1.065 (0.778,1.457)
**Ovary**			
Control	1,551	0.18874	1
AA group	272	0.18268	0.977 (0.859,1.112)
AT/AU group	35	0.22636	1.107 (0.792,1.548)
**Uterus**			
Control	1,100	0.13383	1
AA group	167	0.11214	0.849 (0.721,0.999)
AT/AU group	29	0.18753	1.262 (0.872,1.825)
**Testis**			
Control	160	0.019082	1
AA group	24	0.015893	0.837 (0.545,1.285)
AT/AU group	4	0.023975	1.24 (0.46,3.344)
**Prostate**			
Control	2,091	0.24953	1
AA group	474	0.31412	1.259 (1.139,1.391)
AT/AU group	61	0.36592	1.243 (0.963,1.604)
**Bladder**			
Control	1,116	0.067212	1
AA group	243	0.081022	1.216 (1.059,1.397)
AT/AU group	26	0.080872	1.024 (0.694,1.512)
**Nerve**			
Control	923	0.055587	1
AA group	181	0.060348	1.087 (0.927,1.275)
AT/AU group	25	0.077766	1.287 (0.865,1.916)
**Skin**			
Control	212	0.012766	1
AA group	42	0.014001	1.094 (0.785,1.524)
AT/AU group	2	0.00622	0.417 (0.103,1.677)
**Lymphoma**			
Control	1,295	0.07799	1
AA group	234	0.07802	1.003 (0.873,1.153)
AT/AU group	37	0.1151	1.361 (0.981,1.887)
**Multiple myeloma**			
Control	351	0.021136	1
AA group	68	0.022669	1.089 (0.84,1.412)
AT/AU group	4	0.01244	0.483 (0.18,1.296)
**Leukemia**			
Control	826	0.049744	1
AA group	150	0.050009	1.006 (0.845,1.197)
AT/AU group	15	0.046652	0.879 (0.528,1.466)

Abbreviation: IR = incidence rate.

^a^Multivariate Cox’s regression analyses were adjusted for age and gender; diabetes mellitus, hypertension, and dyslipidemia status; and income level.

^b^The AA group included patients with all types of alopecia areata except alopecia totalis/ alopecia universalis.

^c^The AT/AU group included patients with alopecia totalis (ICD-10 L63.0) and alopecia universalis (ICD-10 L63.1).

## Discussion

To our knowledge, this is the first study to investigate the total cancer risk in alopecia patients using a population-based approach. We found that the total cancer risk was increased in alopecia patients compared with age- and sex- matched controls. This was attributable to an increase in the risks of thyroid, prostate, and bladder cancer depending on the alopecia subtype. In particular, the risk of thyroid cancer was higher in the AT/AU group than in the age- and sex- matched control group or the AA group.

Alopecia is a hair-loss disorder associated with inflammation and an autoimmune response^5^, affecting an estimated 4.5 million people in the United States^6^. AA is thought to be a TH1-mediated autoimmune disease in which the hair follicle loses its status as an immunoprivileged site, resulting in perifollicular CD8+ cytotoxic T cell infiltration followed by elevated IFN-γ production^7^. Genetic polymorphisms in genes encoding cytokines affect their transcriptional levels, associated with inter-individual variations in cytokine production, thus influencing the outcomes of cancers and autoimmune diseases^8^. Lew *et al*. reported that the single-nucleotide polymorphism IL17RA (rs879577) was significantly enriched in AA patients^9^. In addition, a significant association between the intron 3VNTR polymorphism of the IL-4 gene and AA susceptibility was reported in a Turkish population^10^. Kalkan *et al*. found that the AA risk was associated with the FAS-670 A/G and the FAS ligand (FASLG)-124 A/G polymorphisms^11^. Recently, Tanemura *et al*. suggested that AA was a Th17-cell-associated autoimmune disease caused by infiltration of CD4+ expressing IL-17A, and Th17 cells, around hair follicles^12^. Additionally, low serum 25-hydroxyvitamin D [25(OH)D] levels were suggested to contribute to AA pathogenesis^13^.

We found that the risk of thyroid cancer was higher in AA patients than the general population. Chu *et al*. performed a nationwide population-based study in Taiwan and found a relationship between AA and thyroid diseases^14^. In addition, recent studies have shown that AA is associated with autoimmune thyroiditis, but the causative AA subtype was not identified, and thyroid autoantibodies were absent^15,16^. Sun *et al*. reported that the risk of thyroid cancer was increased in females with alopecia, but found no association with AA in general, or a particular AA subtype^4^. Although no association between AA and thyroid cancer was noted in previous studies, it is significant that our present large-scale study revealed such a relationship, most notably in the AT/AU group.

The association between AA and thyroid cancer may be attributable to shared pathological features. Chronic inflammation increases the long-term risk of cancer of the thyroid, prostate, and bladder^17^. FAS and FASLG are pro-apoptotic proteins playing major roles in both cancer development and that of various diseases of the immune system^18^. Reduced FAS expression and/or increased FASLG expression facilitate tumor development and progression by inhibiting tumor cell apoptosis or by inducing immune cell apoptosis. Recently, FAS and FASLG polymorphisms have been reported in various cancers and alopecia^11,19^. Additional mechanism studies including FAS and FASLG are needed to define the risk of thyroid cancer in AT/AU patients.

We found that AA was associated with an increased risk of prostate cancer (HR, 1.259; 95% CI, 1.139–1.391) compared with AT/AU. Few previous reports have sought associations between AA and prostate cancer. Even the suggested association between androgenic alopecia and prostate cancer remains unclear^20–23^. However, androgen levels is suggested influence the pathogenesis of prostate cancer^24^. Taylor *et al*. suggested a relationship between a vitamin D receptor gene polymorphism and prostate cancer^25^. Recently, Conic *et al*. found that AA was associated with vitamin D deficiency and increased androgen levels, as are thyroid disease, anemia, and eczema^26^. Thus, increased androgen levels and a vitamin D deficiency may contribute to the pathogenesis of both diseases. Another shared mechanism may be the pathway involving Th17 cells and IL-17. Zhang *et al*. suggested that IL-17 promoted the initiation and growth of prostate cancer, and that the IL-17-MMP7 pathway was involved in prostatic intraepithelial neoplasia prior to the development of prostate cancer^27^. However, these mechanisms do not adequately explain the relationship between AA and prostate cancer.

Moreover, we found that AA was associated with an increased risk of bladder cancer (HR, 1.216; 95% CI, 1.059–1.397). Androgen-mediated signaling by the androgen receptor is known to play roles in bladder and urothelial cancer^28^. Th17 and Treg cells (especially the former) were involved in the development and progression of bladder cancer^29^. These mechanisms partially overlap with those of prostate cancer (described above). However, it remains difficult to completely explain the associations between alopecia and prostate/bladder cancer; further studies are needed.

In addition, we found that the incidence of skin cancer was not increased in AA patients, consistent with previous studies^30,31^. Miller *et al*. found that the risks of non-melanoma skin cancers (basal cell carcinoma and squamous cell carcinoma), and melanoma, were not increased among AA patients in Cleveland (OH, USA)^30^. Our results also showed that the risk of skin cancer was not increased in AT/AU patients.

Our study had certain limitations. We lacked information on the numbers of alopecia lesions and their treatment, lifestyles, smoking and drinking status, and the levels of physical activity. Also, the ages at onset of alopecia and various cancers differ; our follow-up duration may have been inadequate. Despite these limitations, we found that a large-scale analysis of national data revealed that the risks of thyroid, prostate, and bladder cancer increased depending on the alopecia subtype. Further basic and clinical studies are required. Regular follow-up in terms of thyroid and urinary tract cancers (prostate and bladder cancers) may be necessary for alopecia patients.

## Materials and Methods

### Ethics approval

This study was approved by the Institutional Review Board of the Korean NHIS (no. NHIS-2017–1–138). The study design was approved by the Ethics Committee of Seoul St. Mary’s Hospital, the Catholic University of Korea (approval no. KC17ZESI0312) and followed all relevant principles of the Declaration of Helsinki.

### Data sources

We used the NHIS database, which contains medical information on almost all Koreans. The NHIS manages all health-related data, including checkup results, treatment details, long-term care of the elderly, institutional data, and the frequencies of cancers and rare diseases. The NHIS uses the comprehensive database of the Health Insurance and Review Agency (HIRA)^32^. The database contains patient demographics, outpatient histories, diagnoses and comorbidities based on the International Classification of Disease (ICD)−10 codes, and prescription and procedural details^33^. All patients are assigned identification numbers and all data are managed anonymously.

### Study population

We retrieved data on patients who visited clinics or hospitals and received the diagnostic code (ICD-10) for alopecia (L63) more than once in any year from January 2007 to December 2014. Of these, subjects under 20 years of age or with a history of cancer during a 2005–2006 washout period were excluded. Finally, alopecia patients older than 20 years were included (n = 668,604) (Fig. 1). The control group (n = 3,343,020) was randomly selected at a 1:5 ratio; this group contained matched age- and sex-stratified subjects who were not treated for alopecia during the same period. In addition, we divided alopecia patients into two groups, as follows: 1) an AA group (ICD-10 codes L63.8 and L63.9) including all types of AA except AT/AU (ICD-10 L63); and, 2) an AT/AU group (ICD-10 codes L63.0 and L63.1).

Cancer information was also extracted from the NHIS database; we sought incident cancer reports to 2015. The Korean government maintains records of patients with cardiovascular and cerebrovascular diseases, cancers, and rare incurable diseases (RIDs), and supports such patients financially. We used the cancer code (ICD-10 C00-C96).

### Statistical analysis

Baseline characteristics are presented as means with standard deviations or as numbers with percentages. We calculated cancer incidence rates (in 1,000 person-years) by dividing the number of incident cases by the total follow-up period. Cox’s proportional hazards regression analysis was used to evaluate the association between alopecia presence and subtype, and cancer incidence. Overall and site-specific cancer risks of AA or AT/AU patients compared to those of the age- and sex-matched control population were expressed as hazard ratios (HRs) with 95% confidence intervals (CIs). Model 1 was adjusted for age, gender, and income level; and diabetes mellitus, hypertension, and dyslipidemia status. To avoid temporal bias, we matched the date of alopecia diagnosis with the dates of registration of control subjects. The cumulative cancer incidences by alopecia presence and subtype were calculated using Kaplan–Meier curves, and the log-rank test was employed to analyze differences among the groups. All data were analyzed with the aid of SAS version 9.4 (SAS Institute, Cary, NC, USA) and R version 3.1.0 (the R Foundation for Statistical Computing, Vienna, Austria, http://www.R-project.org) software.
